# Polyunsaturated Fatty Acids: New Molecular Mechanisms and Nutritional Therapeutic Challenges

**DOI:** 10.3390/nu17030588

**Published:** 2025-02-06

**Authors:** Dominique Delmas, Virginie Aires

**Affiliations:** 1 UFR of Heatlh Sciences, Université Bourgogne Europe, F-21000 Dijon, France; 2 INSERM Research Center U1231—Therapies and Immune Response in Cancers Team, Bioactive Molecules and Health Research Group, F-21000 Dijon, France; 3 Centre de Lutte contre le Cancer Georges François Leclerc Center, F-21000 Dijon, France; 4 Inserm US58 Biologie Santé Dijon (BioSanD), F-21000 Dijon, France

The scientific exploration of polyunsaturated fatty acids (PUFAs) continues to unveil their profound impact on human health. These essential nutrients, including omega-3 and omega-6 fatty acids, play pivotal roles in cellular function, lipid metabolism, and inflammatory responses. Yet, their intricate molecular mechanisms and therapeutic potential present both opportunities and challenges that this Special Issue seeks to address. Recent studies have deepened our understanding of PUFAs’ molecular pathways, revealing their nuanced roles in gene expression and lipid metabolism. Linoleic acid (LA), an omega-6 PUFA, exemplifies this complexity. Research highlights its dual nature—while it is a precursor to arachidonic acid (AA) and inflammatory mediators, LA independently influences cholesterol biosynthesis, lipid homeostasis, and keratinocyte differentiation. These findings suggest that LA’s effects extend beyond its metabolic conversion, with implications for sebaceous gland function and skin health. Similarly, omega-3 fatty acids such as eicosapentaenoic acid (EPA) and docosahexaenoic acid (DHA) are celebrated for their anti-inflammatory and neuroprotective properties. The interplay between these fatty acids and their cellular targets underscores their potential in mitigating chronic diseases, from cardiovascular ailments to neurodegenerative disorders. However, as research delves deeper, the balance between omega-6 and omega-3 PUFAs emerges as a critical determinant of health outcomes, calling for a re-evaluation of dietary recommendations.

The therapeutic application of PUFAs extends across diverse medical fields. Advances in lipidomic profiling and molecular biology have paved the way for targeted interventions. For instance, topical applications of LA have shown promise in treating acne through the modulation of lipid production and inflammatory responses in sebocytes. Meanwhile, the anti-inflammatory properties of omega-3 PUFAs are being harnessed in therapies for rheumatoid arthritis and inflammatory bowel disease.

Despite these advancements, challenges persist. The bioavailability of PUFAs, influenced by dietary sources and metabolic pathways, remains a barrier to their effective utilization. Innovative delivery systems, including nanotechnologies and encapsulation methods, are being explored to enhance PUFA stability and absorption. Moreover, the potential side effects of PUFA-derived metabolites, such as pro-inflammatory eicosanoids from AA, warrant careful consideration in therapeutic design.

This Special Issue is dedicated to the current knowledge surrounding the molecular mechanism of PUFAs, as well as the latest scientific advances in various domains including metabolism, diseases, and clinical applications.

First of all, Eva C. Hermans and colleagues explore the protective effects of n-3 long-chain PUFAs against neonatal hypoxic–ischemic brain injury, presenting insights into their neuroprotective properties and their limitations when combined with stem cell therapy Contribution 1. Secondly, Heike Speckmann and colleagues highlight the role of synbiotic formulations combining Bacillus megaterium DSM 32963 with n-3 PUFA salts in elevating pro-resolving lipid mediators, highlighting their implications for inflammation resolution Contribution 2. In another article, Christopher Kim and colleagues examine the therapeutic potential of PUFAs in enhancing recovery following post-transfusion injuries, shedding light on their complex roles in hematological health Contribution 3. Furthermore, Dóra Kovács and colleagues investigate the regulatory effects of PUFAs on sebocyte activity and lipid production, with a focus on implications for skin health; they highlight that linoleic acid may have a complex effect both as a precursor of arachidonic acid and independently by modifying the levels of other biologically active lipids Contribution 4. Additionally, Malgorzata Sidorkiewicz reviews the interplay between dietary PUFA supplementation and systemic inflammation, where she analyzes the role of PUFAs and PUFA derivatives in health-related effects, considering both confirmed benefits and newly arising controversies Contribution 5. Finally, Charalambos Michaeloudes and colleagues review the differential effects of EP and DHA on cell function in the context of atherosclerosis. Specifically, they propose steps to improve clinical and basic experimental study design to improve supplement composition optimization Contribution 6.

As this Special Issue demonstrates, the study of PUFAs is at an exciting juncture. The integration of omics technologies and advanced modeling techniques is poised to decode their multifaceted roles in health and diseases. However, translating these insights into practical health strategies requires a collaborative effort among researchers, clinicians, and policymakers.

Future research must address the following key questions: How can we optimize PUFA supplementation to balance their benefits and risks? What are the long-term implications of PUFA-based therapies on systemic inflammation and metabolic health? How can dietary interventions be tailored to individual genetic and metabolic profiles?

In conclusion, PUFAs offer a compelling glimpse into the interplay between diet, molecular biology, and health. By embracing the challenges and opportunities they present, we move closer to unlocking their full therapeutic potential. This Special Issue is a testament to the ongoing quest to harness these essential nutrients for a better efficiency.

## List of Contributions

1. Hermans, E.C.; van Gerven, C.C.E.; Johnsen, L.; Tungen, J.E.; Nijboer, C.H.; de Theije, C.G.M. Dietary LPC-Bound n-3 LCPUFA Protects against Neonatal Brain Injury in Mice but Does Not Enhance Stem Cell Therapy. *Nutrients* **2024**, *16*, 2252. https://doi.org/10.3390/nu16142252.
2. Speckmann, B.; Wagner, T.; Jordan, P.M.; Werz, O.; Wilhelm, M.; Tom Dieck, H.; Schon, C. Synbiotic Bacillus megaterium DSM 32963 and n-3 PUFA Salt Composition Elevates Pro-Resolving Lipid Mediator Levels in Healthy Subjects: A Randomized Controlled Study. *Nutrients* **2024**, *16*, 1354. https://doi.org/10.3390/nu16091354.
3. Kim, C.Y.; Larsen, H.J.; Spitalnik, S.L.; Hod, E.A.; Francis, R.O.; Hudson, K.E.; Gordy, D.E.; Stone, E.F.; Peltier, S.; Amireault, P.; et al. Low-Dose Dietary Fish Oil Improves RBC Deformability without Improving Post-Transfusion Recovery in Mice. *Nutrients* **2023**, *15*, 4456. https://doi.org/10.3390/nu15204456.
4. Kovacs, D.; Camera, E.; Poliska, S.; Cavallo, A.; Maiellaro, M.; Dull, K.; Gruber, F.; Zouboulis, C.C.; Szegedi, A.; Torocsik, D. Linoleic Acid Induced Changes in SZ95 Sebocytes-Comparison with Palmitic Acid and Arachidonic Acid. *Nutrients* **2023**, *15*, 3315. https://doi.org/10.3390/nu15153315.
5. Sidorkiewicz, M. The Cardioprotective Effects of Polyunsaturated Fatty Acids Depends on the Balance Between Their Anti- and Pro-Oxidative Properties. *Nutrients* **2024**, *16*, 3937. https://doi.org/10.3390/nu16223937.
6. Michaeloudes, C.; Christodoulides, S.; Christodoulou, P.; Kyriakou, T.C.; Patrikios, I.; Stephanou, A. Variability in the Clinical Effects of the Omega-3 Polyunsaturated Fatty Acids DHA and EPA in Cardiovascular Disease-Possible Causes and Future Considerations. *Nutrients* **2023**, *15*, 4830. https://doi.org/10.3390/nu15224830.

